# Supplementary material for: Transcriptomic Profiles from Stereo-EEGs May Reflect the Local Brain Cell Microenvironment in Human Epilepsy
Source: Biomolecules. 2025 Dec 2;15(12):1684. doi: 10.3390/biom15121684 (PMC12730889; doi:10.3390/biom15121684)
Supplement: Supplementary file 1 [file biomolecules-15-01684-s001.zip › biomolecules-3976875-supplementary.pdf]

biomolecules

MDPI

Supporting Information

Transcriptomic profiles from stereo-EEGs may reflect the local brain cell microenvironment in human epilepsy

Julian Larkin, Anuj Kumar Dwivedi, Arun Mahesh, Albert Sanfeliu, Kieron J. Sweeney, Donncha F. O'Brien, Vijay K. Tiwari, Peter Widdess-Walsh, and David C. Henshall

Supplementary Table S1: Clinical characteristics of patients

	Age	Sex	Epilepsy Classification	Epilepsy Duration (years)	No. ASMs	Surgical Outcome
**Patient A**	49	Male	**Clinical:** Frontal lobe epilepsy<br>**Imaging:** Focal cortical dysplasia<br>**Pathology:** FCD IIa	34	10	1A
**Patient B**	40	Female	**Clinical:** Temporal lobe epilepsy<br>**Imaging:** Non-lesional<br>**Pathology:** Chaslin's subpial gliosis/non-specific	8	7	2A
**Patient C**	24	Female	**Clinical:** Right hemisphere epilepsy<br>**Imaging:** Right hemi-atrophy, gliosis<br>**Pathology:** Gliosis, minimal T-cell infiltrate, perivascular chronic inflammation	8	12	1A

Supplementary Table S2: Top 20 GO Enriched Processes, Patient B vs C (upregulated)

ID	Description	GeneRatio	BgRatio	RichFactor	FoldEnrichment	zScore	pvalue	p.adjust	qvalue	Count
**GO:0051783**	regulation of nuclear division	13/284	151/18986	0.08609272	5.7554799	7.22970299	4.53988579318626e-07	0.00158397	0.00134524	13
**GO:0000280**	nuclear division	22/284	451/18986	0.04878049	3.26107867	5.98863843	1.30614837769494e-06	0.0018843	0.00160031	22
**GO:0007088**	regulation of mitotic nuclear division	11/284	121/18986	0.09090909	6.07746479	6.90449064	2.058294633187e-06	0.0018843	0.00160031	11
**GO:0002476**	antigen processing and presentation of endogenous peptide antigen via MHC class Ib	5/284	17/18986	0.29411765	19.6623861	9.48614714	3.86109282371329e-06	0.0018843	0.00160031	5
**GO:0002484**	antigen processing and presentation of endogenous peptide antigen via MHC class I via ER pathway	5/284	17/18986	0.29411765	19.6623861	9.48614714	3.86109282371329e-06	0.0018843	0.00160031	5
**GO:0002428**	antigen processing and presentation of peptide antigen via MHC class Ib	5/284	18/18986	0.27777778	18.5700313	9.19006243	5.28089050143574e-06	0.0018843	0.00160031	5
**GO:0140014**	mitotic nuclear division	16/284	282/18986	0.05673759	3.79302767	5.82308313	5.80177393094869e-06	0.0018843	0.00160031	16
**GO:0051784**	negative regulation of nuclear division	8/284	67/18986	0.11940299	7.98234181	7.05520866	6.89811029668924e-06	0.0018843	0.00160031	8

**GO:0000070**	mitotic sister chromatid segregation	13/284	194/18986	0.06701031	4.47978075	6.00325358	7.51547121139626e-06	0.0018843	0.00160031	13
**GO:0007094**	mitotic spindle assembly checkpoint signaling	7/284	49/18986	0.14285714	9.55030181	7.38489525	7.81029290391766e-06	0.0018843	0.00160031	7
**GO:0071173**	spindle assembly checkpoint signal-ing	7/284	49/18986	0.14285714	9.55030181	7.38489525	7.81029290391766e-06	0.0018843	0.00160031	7
**GO:0071174**	mitotic spindle checkpoint signaling	7/284	49/18986	0.14285714	9.55030181	7.38489525	7.81029290391766e-06	0.0018843	0.00160031	7
**GO:0098813**	nuclear chromosome segregation	17/284	324/18986	0.05246914	3.50767258	5.61028195	8.35925942936167e-06	0.0018843	0.00160031	17
**GO:0035115**	embryonic forelimb morphogenesis	6/284	33/18986	0.18181818	12.1549296	7.90324174	8.37962046705594e-06	0.0018843	0.00160031	6
**GO:0031577**	spindle checkpoint signaling	7/284	50/18986	0.14	9.35929578	7.29341652	8.96658022427903e-06	0.0018843	0.00160031	7
**GO:0033046**	negative regulation of sister chroma-tid segregation	7/284	51/18986	0.1372549	9.17578017	7.20447077	1.02613221486692e-05	0.0018843	0.00160031	7
**GO:0033048**	negative regulation of mitotic sister chromatid segregation	7/284	51/18986	0.1372549	9.17578017	7.20447077	1.02613221486692e-05	0.0018843	0.00160031	7
**GO:0045841**	negative regulation of mitotic meta-phase/anaphase transition	7/284	51/18986	0.1372549	9.17578017	7.20447077	1.02613221486692e-05	0.0018843	0.00160031	7

**GO:2000816**	negative regulation of mitotic sister chromatid separation	7/284	51/18986	0.1372549	9.17578017	7.20447077	1.02613221486692e-05	0.0018843	0.00160031	7
**GO:0000819**	sister chromatid segregation	14/284	235/18986	0.05957447	3.98267905	5.66955122	1.29241046075847e-05	0.00211209	0.00179376	14

Supplementary Figure S1: Expression of housekeeping genes and HLA-related genes across patients

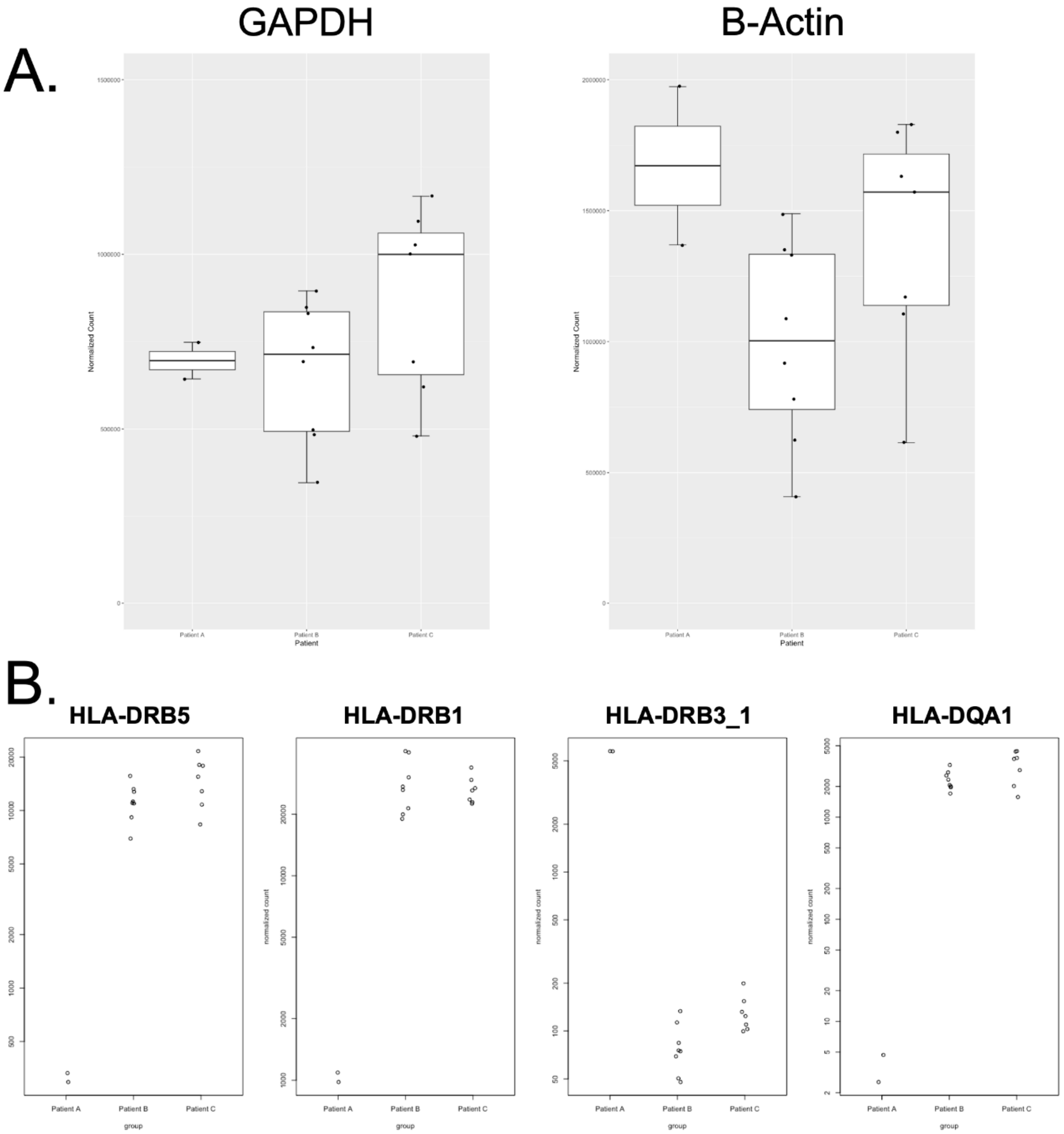

Expression of housekeeping genes and HLA-related genes across patients: A. *GADPH* and B-Actin expression does not significantly differ across samples between patients. B. *HLA-DRB5*, *HLA-DRB1*, *HLA-DRB3_1* and *HLA-DQA1* differ significantly between patients.

Supplementary Figure S2: Expression of anatomical region-specific transcripts between the cingulate and all other structures

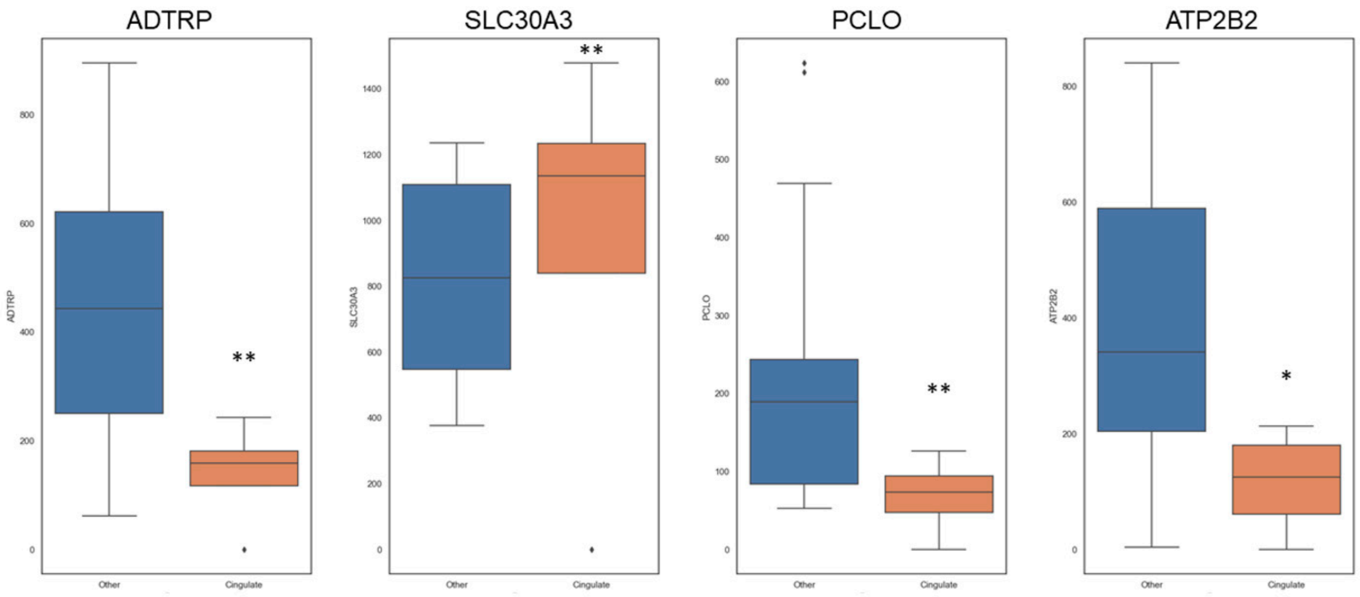

Expression of anatomical region-specific transcripts between the cingulate and all other structures. *ADTRP* (p=0.00104), *PCLO* (p=0.0099), and *ATP2B2* (p=0.01) expression is significantly lower in the cingulate than in all other structures. *SLC30A3* expression is higher in the cingulate compared to other structures (p=0.00444). Student's unpaired t-test, two-tailed.

Supplementary Figure S3: Normalised counts for selected genes across seizure network

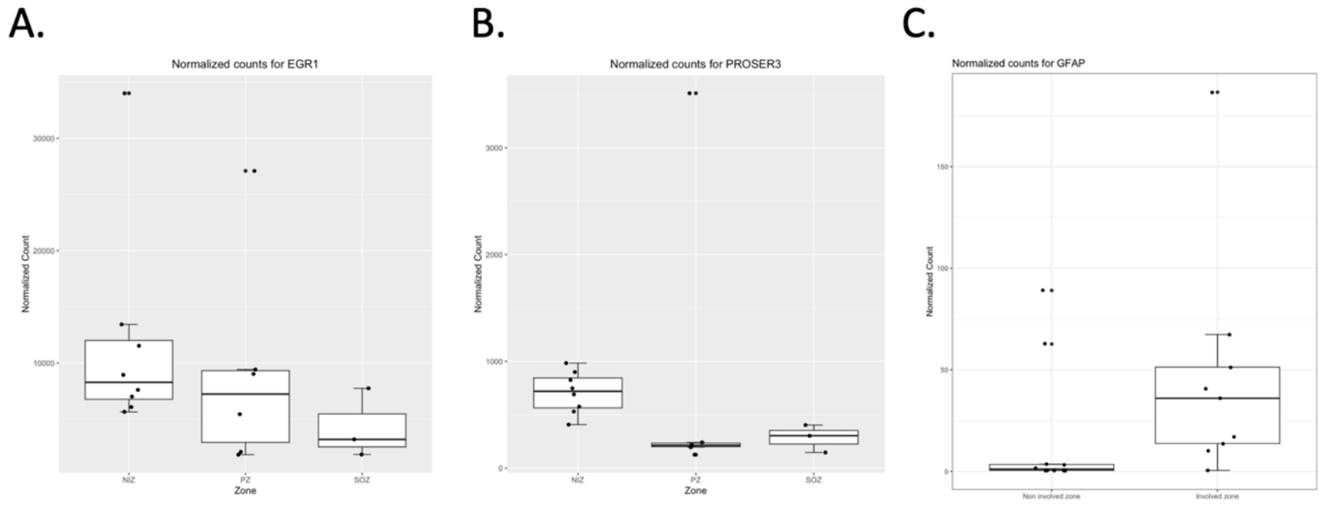

Normalised counts for selected genes across seizure network: A. Boxplot showing a non-significant decrease of EGR1 expression across the non-involved zone (NIZ), propagation zone (PZ), and seizure onset zone (SOZ). B. PROSER3 counts are significantly lower in the PZ and SOZ compared to the NIZ (p=0.0476). C. GFAP expression in the NIZ compared to the ictogenic network strucutres (PZ and SOZ).
